# Effects of DACT1 methylation status on invasion and metastasis of nasopharyngeal carcinoma

**DOI:** 10.1186/s40659-019-0238-3

**Published:** 2019-06-10

**Authors:** Ju-Hong Yang, Lie-Kun Lin, Song Zhang

**Affiliations:** 1 Central Laboratory, University of Chinese Academy Sciences-Shenzhen Hospital, Song Bai Road 4253#, Shenzhen, 518106 Guang Dong People’s Republic of China; 2 Department of Otolaryngology, University of Chinese Academy Sciences-Shenzhen Hospital, Song Bai Road 4253#, Shenzhen, 518106 Guang Dong People’s Republic of China

**Keywords:** DACT1 gene, Nasopharyngeal carcinoma, CNE2 cell line, Invasion, Metastasis, 5-Aza-2-deoxycytidine

## Abstract

**Background:**

The purpose of the present study was to investigate the role of the methylation status of the DACT1 gene on the invasion and metastasis of nasopharyngeal carcinoma cells.

**Methods:**

The levels of methylation and expression of the DACT1 gene in nasopharyngeal carcinoma tissues and CNE2 cells were determined by methylation-specific PCR and RT-PCR, respectively. CNE2 cells were treated with 5-aza-2-deoxycytidine, and the variation in the methylation status of the DACT1 gene was detected, as well as the influence of methylation on invasiveness of nasopharyngeal carcinoma cells.

**Results:**

The DACT1 gene was hyper-methylated in 44 of 62 cases of nasopharyngeal carcinoma. The DACT1 gene was hyper-methylated in 32 of 38 cases of nasopharyngeal carcinoma with lymph node metastasis, and the DACT1 gene was hyper-methylated in 7 of 24 cases of nasopharyngeal carcinoma without lymph node metastasis. The DACT1 mRNA level was weakly expressed or not expressed in all nasopharyngeal carcinoma tissues with hyper-methylated DACT1 genes; however, the DACT1 mRNA level was highly expressed in nasopharyngeal carcinoma tissues with low expression of the methylated DACT1 gene. The DACT1 gene was hyper-methylated and not expressed in CNE2 cells that did not have 5-aza-2-deoxycytidine treatment. After 5-aza-2-deoxycytidine treatment, the DACT1 gene was demethylated and the expression of DACT1 was restored. Moreover, the invasion ability was inhibited in CNE2 cells treated with 5-aza-2-deoxycytidine.

**Conclusion:**

The expression of DACT1 was related to the methylation status. High expression of DACT1 may inhibit the invasion and metastasis of nasopharyngeal carcinoma cells.

## Background

DNA methylation is a major property of gene epigenetics. The occurrence and development of various tumors are related to gene methylation. Inactivation of hyper-methylation in the promoter region of the tumor suppressor genes plays an important role in tumorigenesis and development of tumors. Activation of hypo-methylation of the proto-oncogene also plays an important role in the development of some malignant tumors. The Dapper1/Dpr1 (DACT1) gene is a tumor-associated gene that plays an important role in the regulation of tumor cell growth, proliferation, invasion, and metastasis [1]. Yang et al. [2] have shown that DACT1 expression is related to pathologic grade, size, invasion, and metastasis of non-small cell lung cancers [2].

Nasopharyngeal carcinoma (NPC) is a common malignant tumor with a high incidence in China, especially in Guangdong Province. A prominent clinical feature of NPC is early cervical lymph node metastasis. Indeed, the main reason for the poor prognosis in patients with NPCs is invasion and metastasis; however, the mechanism underlying metastasis is not clear. In the present study we determined the expression and methylation status of DACT1 in NPC tissues. The methylation status and expression of the DACT1 gene was determined before and after 5-aza-2-deoxycytidine treatment, as well as the invasive ability of NPC cells. The function of DACT1 was analyzed with respect to the occurrence, development, and metastasis of NPC.

## Methods

### Clinical information

The specimens included 62 cases of NPC tissues and 19 cases of chronic inflammation tissues of the nasopharynx. All the specimens were collected from the patients in our hospital with suspicious nasopharyngeal neoplasms. None of the patients received radiotherapy or chemotherapy before biopsy. Among the 62 patients with NPCs, there were 44 males and 18 females. According to the 8th edition of the UICC TNM staging system, there were 14 T1 cases, 19 T2 cases, 18 T3 cases, and 11 T4 cases. There were 38 cases with lymph node metastases and 24 cases had no lymph node metastasis.

### Cell culture

CNE2 cells were purchased from the Shanghai Institute of Cell Biology (Chinese Academy of Life Science). The cells were cultured in 1640 medium containing 10% fetal calf serum in 5% CO_2_ at 37 °C. CNE2 cells were digested with trypsin into single cell suspensions in the logarithmic growth phase, then seeded in six-well plates in equal amounts. The next day, the cells in the six-well plates were randomly divided into two groups: one group of cells were treated with 80 μmol/L of 5-aza-2-deoxycytidine as the experimental group; and the other group was used as control group without any drug. After 1 day of drug treatment, the medium was changed for further culture. Two days later, the cells were harvested for subsequent experiments.

### Methylation-specific PCR

After the cells were harvested, the total DNA of each group of cells was extracted using a DNA extraction kit. Then, the bisulfite modification was carried out using a DNA methylation kit. According to the DACT1 gene sequence in GeneBank, the methylated primers and unmethylated primers were designed. The sequences of methylated primers (M) were 5-CGGGATAGTAGTAGTCGGC-3 (S) and 5-CGCTAAAACTACGACCGCG-3 (R). The sequences of unmethylated primer (U) were 5-GTTGGGATAGTAGTAGTTGGT-3 (S) and 5-AAACACTAAAACTACAACCACA-3. The DNA sample in each group of sulfite-modified cells was amplified by two kinds of primers. The annealing temperature was 60 °C (methylation) and 58 °C (unmethylation). 10 μL of the PCR products were taken to be detected in 2% agarose gel electrophoresis, and DACT1 gene methylation was observed.

### RT-PCR

The total RNA of these cells was extracted with TRIzol reagent and reverse transcribed with reverse transcriptase to form the first strand of DNA (cDNA). Two pairs of primers for DACT1 and β-actin were designed according to the gene sequences in GeneBank, and the cDNAs were used as templates for PCR. The DACT1 primers were 5-GGAAGAGGACAGGCTTGGAAAC-3 (S) and 5-GTCCCATTGTTCAGAGAAGGTATC-3 (R). The β-actin primers were 5-CACCCAGCACAATGAAGATCAAGAT-3 and 5-CCAGTTTTTAAATCCTGAGTCAAGC-3. The annealing temperature was 60 °C. After the reaction, 5 μL of the PCR product was obtained for electrophoresis in 1.5% agarose gel and the expression of DACT1 was observed.

### Cell invasion assay (Transwell method)

The control and drug-treated cells were cultured for 24 h and digested to single-cell suspension at a density of 10^5^/mL. A 1 mL cell suspension was centrifuged at 1500*g* for 5 min, and the supernatant was discarded. Two hundred microliters of serum-free medium were added, and mixed well prior to placement in a Transwell chamber. Five hundred microliters of complete medium containing 10% FBS were added to the Transwell plates and the chambers were placed into the plate. The cells were cultured at 37 °C in a 5% CO_2_ incubator for 24 h. Then, the chamber was removed, the medium was washed away with PBS, and stained with crystal violet for 10 min. Then, the surface of the crystal violet was washed away and the cells in the upper chamber were cleaned with a cotton swab and observed under an inverted microscope. The number of cells in each field was recorded randomly in each group and the difference in invasion ability between the two groups was analyzed.

### Statistical analysis

Statistical analysis was performed using statistical software (SPSS 17.0 for Windows). The methylation rate of the DACT1 gene in different T stages of nasopharyngeal carcinomas was analyzed using a Chi-square test. The differences in cell invasive ability were analyzed using an independent samples t-test. A P < 0.05 was considered significantly different.

## Results

**Fig. 1:**
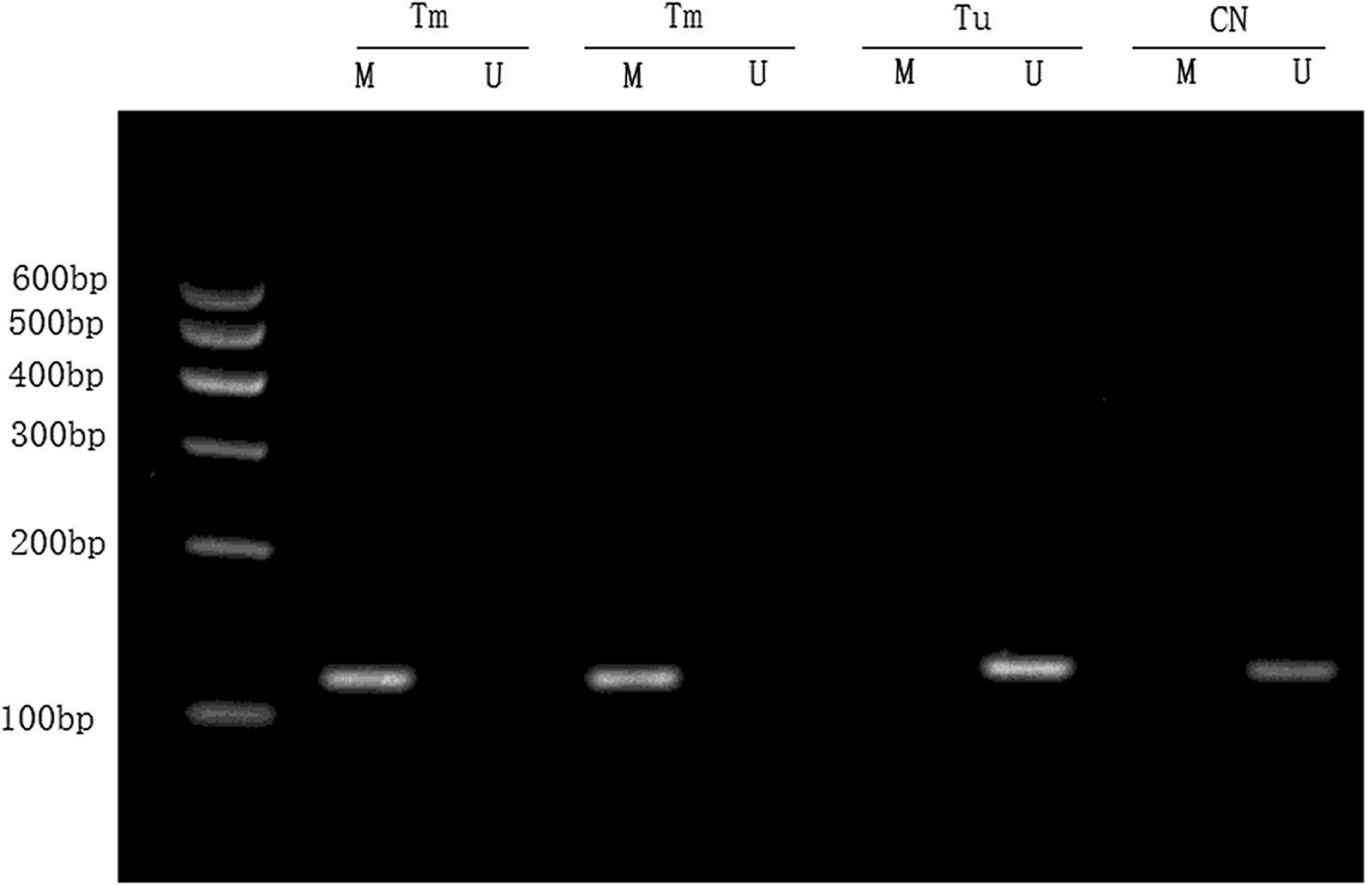
Methylation of the DACT1 gene in nasopharyngeal carcinoma and nasopharyngeal chronic inflammatory tissues. Tm: the nasopharyngeal carcinoma tissue with methylated DACT1 gene; Tu: the nasopharyngeal carcinoma tissue with unmethylated DACT1 gene; CN: chronic nasopharyngeal inflammatory tissue; M: amplification product of methylated primers; U: amplification product with non-methylated primers

The methylation-specific PCR results showed that 10 cases of NPC tissues were T_1_ stage, 13 cases were T_2_ stage, 13 cases were T_3_ stage, and 8 cases were T_4_ stage with hyper-methylation of the DACT1 gene promoter. The Chi-square test showed that the methylation rate of the DACT1 gene was not significantly different in the T_1_, T_2_, T_3_, and T_4_ stages (P > 0.05; Fig. 1). The DACT1 gene promoter was hyper-methylated in 32 of 38 cases of NPC with lymph node metastases; however, the DACT1 gene promoter were hyper-methylated in 7 of 24 cases of NPC without lymph node metastases. There was a statistical difference between the two groups (P < 0.01).

**Fig. 2:**
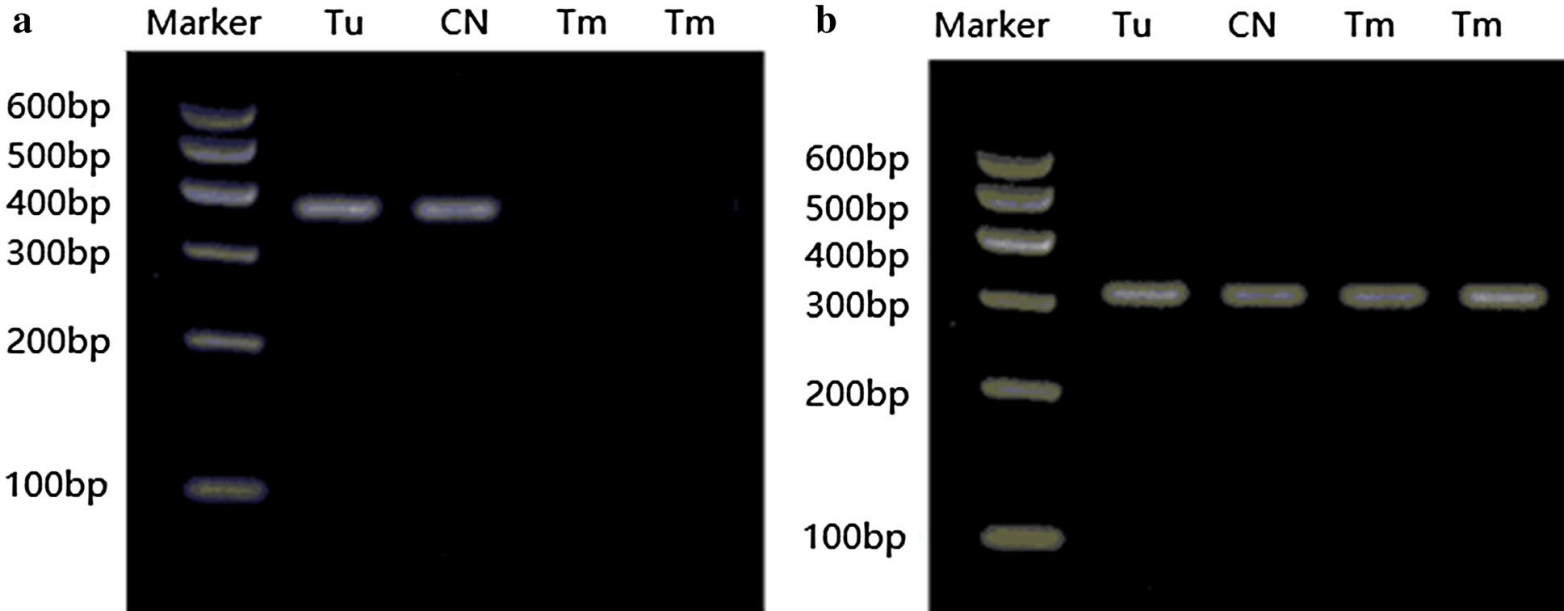
DACT1 mRNA expression in nasopharyngeal carcinoma and nasopharyngeal chronic inflammation tissue. Tm: the nasopharyngeal carcinoma tissue with methylated DACT1 gene; Tu: the nasopharyngeal carcinoma tissue with unmethylated DACT1 gene; CN: chronic nasopharyngeal inflammatory tissue. **a** DACT1 mRNA expression, **b** β-action mRNA expression

The RT-PCR results revealed that there was no DACT1 mRNA expression in all methylated NPCs; however, DACT1 mRNA was highly expressed in unmethylated NPC tissues and the chronic inflammatory tissues in the nasopharynx (Fig. 2).

**Fig. 3:**
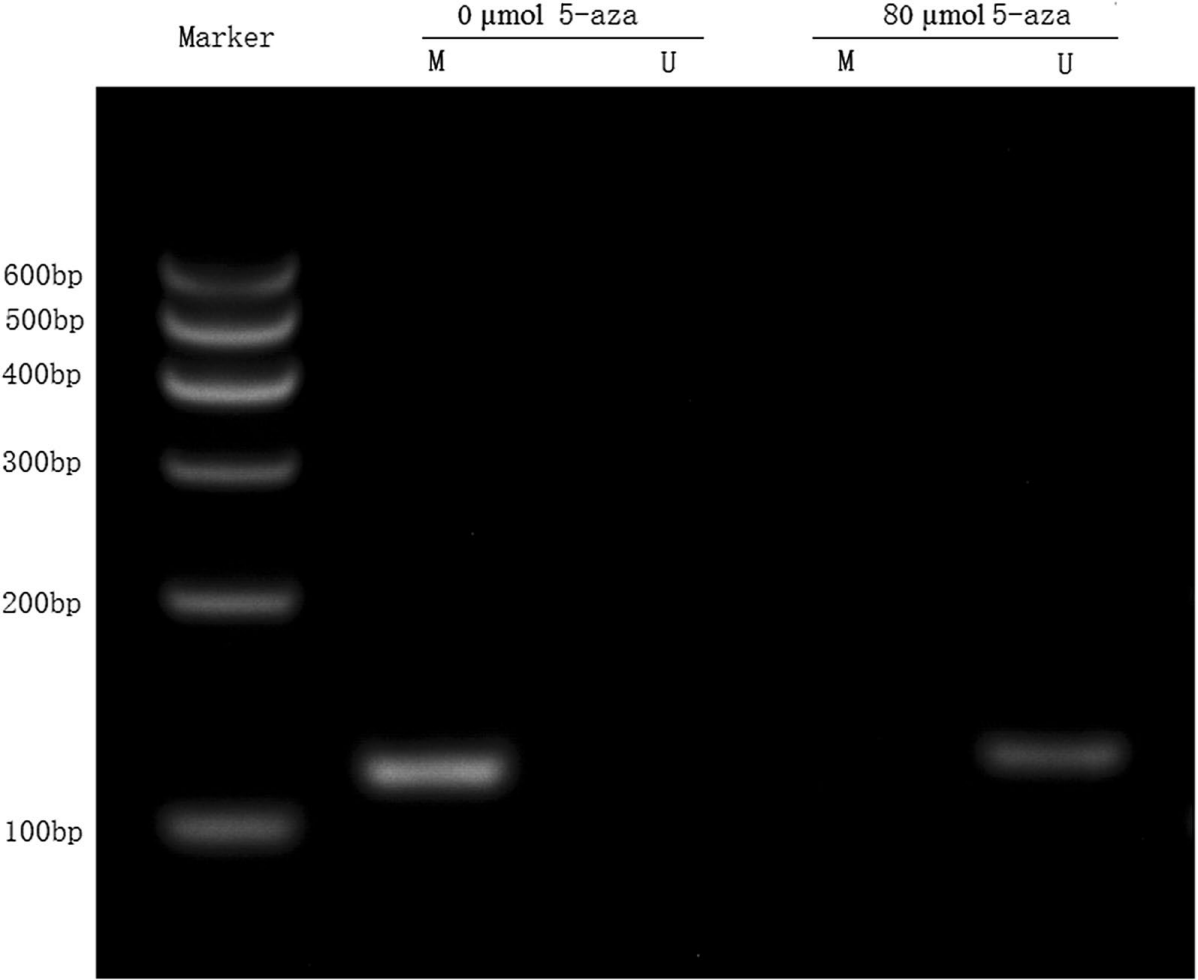
Methylation status of DACT1 gene in CNE2 cells before and after 5-aza-2-deoxycytidine treatment. 0 μmol/L 5-aza: nasopharyngeal carcinoma cells without drug treatment, 80 μmol/L 5-aza, drug concentration was 80 μmol/L in nasopharyngeal carcinoma cells; M: amplification products by methylation primers; U: amplification product by unmethylated primer

**Fig. 4:**
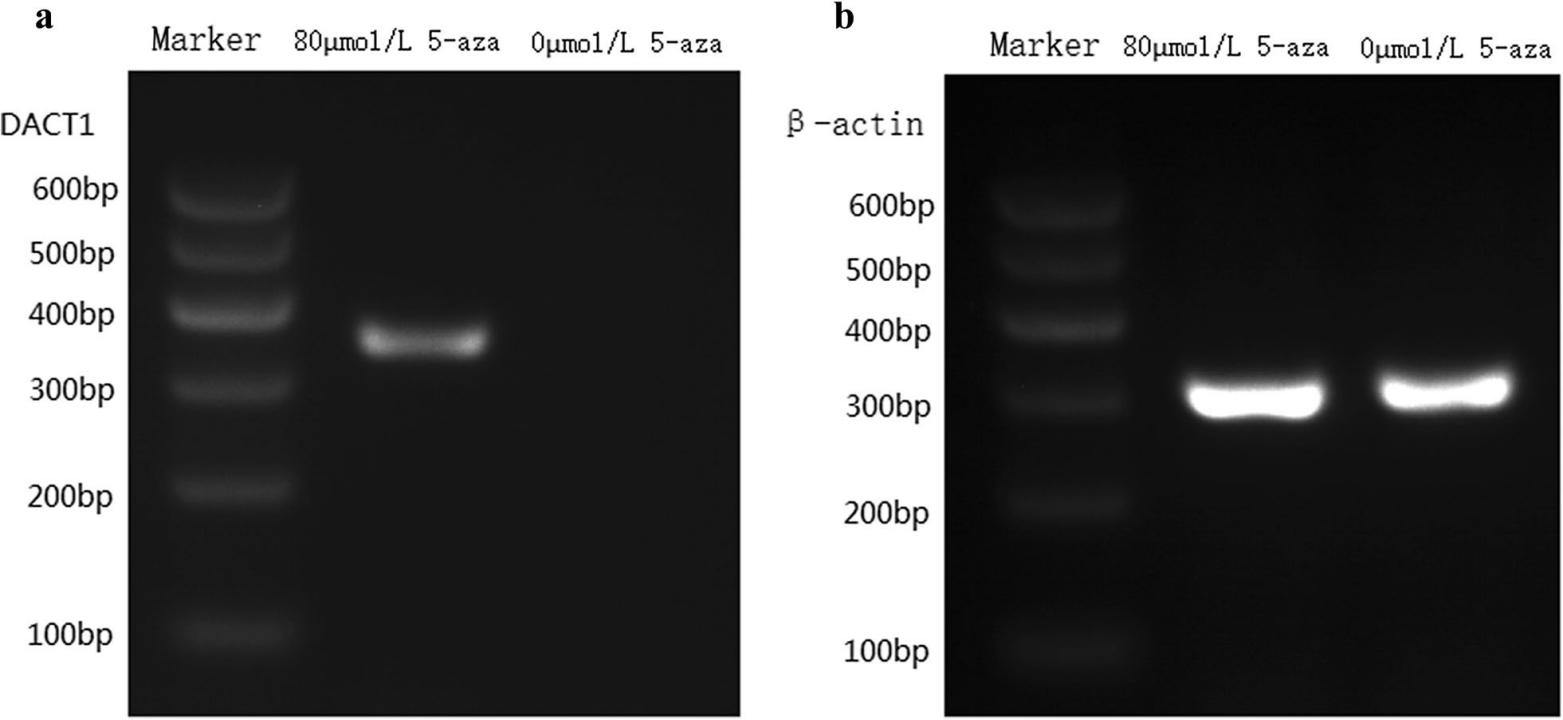
DACT1 mRNA expression in CNE2 cells before and after 5-aza-2-deoxycytidine treatment. **a** The expression of DACT1 mRNA in each tissue. **b** The expression of β-actin mRNA in each tissue. 0 μmol/L 5-aza: nasopharyngeal carcinoma cells without drug treatment, 80 μmol/L 5-aza, drug concentration was 80 μmol/L in nasopharyngeal carcinoma cells

Methylation-specific PCR results showed that the DACT1 gene was hyper-methylated in CNE2 cells without 5-*N*-deoxycytidine treatment, but the DACT1 gene was demethylated in CNE2 cells treated with 5-aza-deoxycytidine (Fig. 3). The RT-PCR results suggested that DACT1 was not expressed in CNE2 cells not treated with 5-azacytidine, whereas the expression of DACT1 was restored in CNE2 cells treated with 5-aza-deoxycytidine (Fig. 4). All the data indicated that 5-aza-deoxycytidine regained the expression of DACT1 gene by changing the methylation status.

**Fig. 5:**
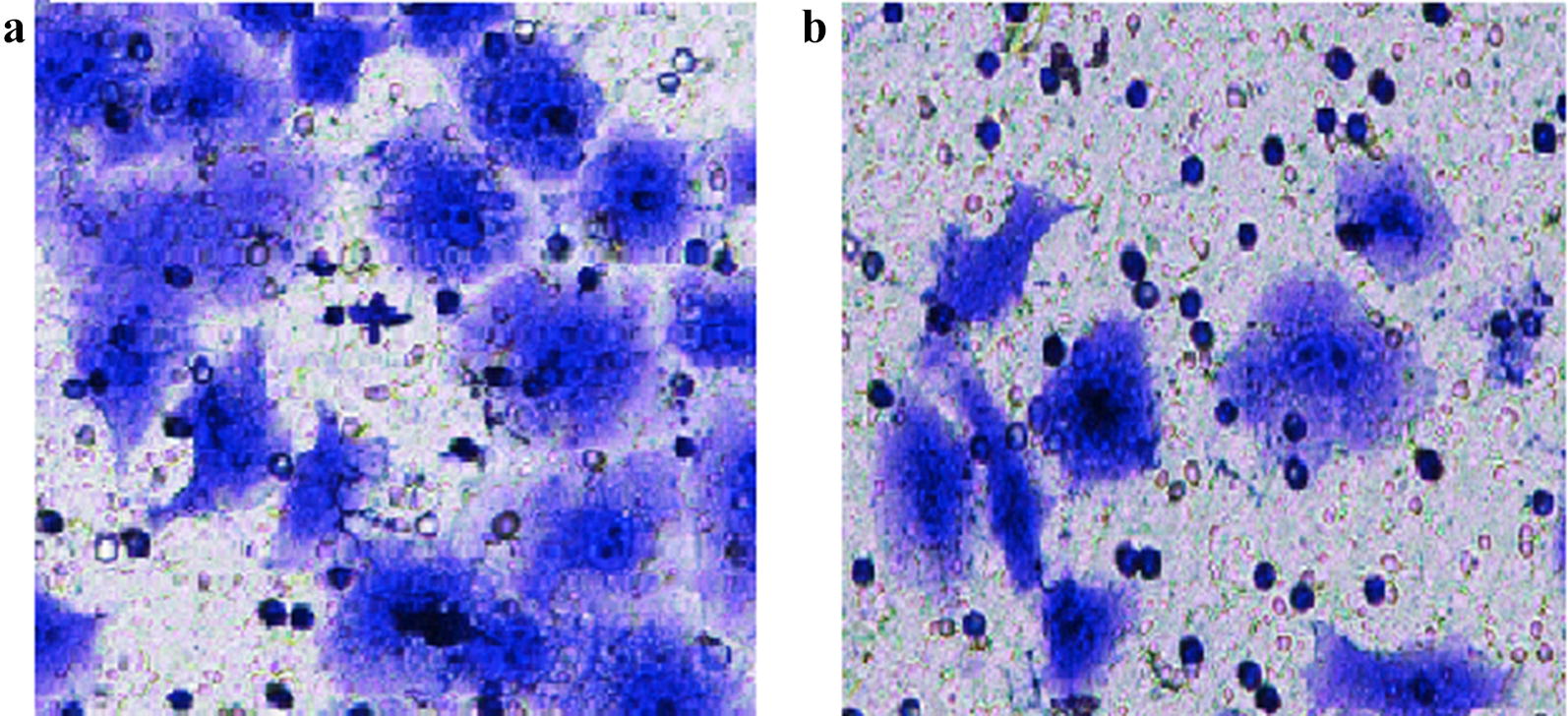
The invasive abilities before and after 5-aza-2-deoxycytidine treatment of CNE2 cells. **a** CNE2 cells without 5-aza-2-deoxycytidine treatment, **b** CNE2 cells with 5-aza-2-deoxycytidine treatment

The Transwell assay results showed that the number of invasive CNE2 cells not treated with 5-aza-2-deoxycytidine (X = 13.50 ± 2.15) was significantly higher than CNE2 cells treated with 5-aza-2-deoxycytidine (X = 8.50 ± 1.08, t = 5.868, P < 0.001; Fig. 5). The results demonstrated that the expression of DACT1 may affect the invasive ability of CNE2 cells.

## Discussion

The DACT1 gene is located at 14q22.3 and encodes a protein with 836 amino acids; there is a leucine-linked domain at the amino terminus and a PDZ linkage at the carboxy terminus [1]. Recent studies have shown that DACT1 is a tumor-related gene that may regulate tumor cell growth, proliferation, invasion, and metastasis by increasing the expression of beta-catenin, affecting the Wnt signaling pathway, and regulating the cell cycle and apoptosis [2]. Wang et al. [3] reported that the loss of DACT1 expression in colon cancer cells may be related to the growth, proliferation, migration, and invasion of cancer cells. Yang et al. [4] reported that low expression of DACT1 in non-small cell lung cancer is associated with a poor prognosis. Moskalev et al. [5] concluded that the high expression of DACT1 in B cell chronic lymphocytic leukemia cells is related to low methylation of the DACT1 gene.

DNA methylation is an important feature of epigenetic modification of the gene, which regulates the transcriptional activity of specific genomic regions under the conditions of a constant DNA sequence and controls the expression of the gene [6]. The CpG island methylation status of a gene promoter is related to the activity of the gene. Hyper-methylation of the CpG island in the promoter region is one of the major causes of gene inactivation. In recent years, many researchers have reported that some tumor suppressor genes are inactivated due to methylation of the promoter region, which may promote tumorigenesis and metastasis [7–9]. Some oncogenes are activated due to promoter demethylation and promote the occurrence and development of tumors [10, 11]. Our previous study showed that the expression of the tumor suppressor gene is related to the methylation status of CpG islands in the promoter region of the genes [12, 13]. The methylation status of these genes is reversible and can be regulated by regulating the activities of enzymes to change the expression of these genes, thereby affecting the growth and invasion of nasopharyngeal and laryngeal cancer cells.

The results of this study showed that the DACT1 gene promoter was hyper-methylated in 44 of 62 cases of NPC. The DACT1 gene promoter was hyper-methylated in 32 of 38 NPC patients with lymph node metastases. Moreover, there was no DACT1 gene expression in methylated NPC tissues. The results showed that the expression of DACT1 in NPC was related to hyper-methylation of the promoter region. It was also confirmed that the DACT1 gene was inactivated by hyper-methylation in the promoter region with 5-aza-deoxycytidine treatment, which can be reactivated by demethylation. The reactivated DACT1 gene inhibited the invasion and metastasis of NPC cells. Guo et al. [14] have found that abnormal methylation of the DACT1 gene was associated with the progress and poor prognosis of esophageal squamous cell carcinoma. Deng et al. [15] found that the number of methylation sites in the CpG island of the DACT1 gene was related to the prognosis of the disease.

## Conclusions

In summary, in NPC tissues and cells, DACT1 gene expression was related to the methylation status, which can be changed by regulating the methylation status and regulate the growth and invasion of tumor cells. DACT1 may be an important indicator for progression and prognosis of NPC in the future.

## Data Availability

The datasets used and/or analyzed during the current study are available from the corresponding author on reasonable request.

## Abbreviations

DACT1 (Dapper): originally identified as an interacting protein for dishevelled (Dvl)
NPC: nasopharyngeal carcinoma
RT-PCR: reverse transcription polymerase chain reaction
